# Temporal trends and risk factors for parathyroidectomy in the Swedish dialysis and transplant population – a nationwide, population-based study 1991 – 2009

**DOI:** 10.1186/1471-2369-15-75

**Published:** 2014-05-08

**Authors:** Shahriar Akaberi, Naomi Clyne, Gunnar Sterner, Bengt Rippe, Eva Reihnér, Philippe Wagner, Rebecca Rylance, Karl Göran Prütz, Martin Almquist

**Affiliations:** 1 Department of Nephrology, Clinical Sciences Lund, Lund University, Alwallhuset, Barngatan 2A, Lund 221 85, Sweden; 2 Department of Nephrology, Clinical Sciences Malmö, Lund University, Lund, Sweden; 3 Department of Molecular Medicine and Surgery, Karolinska Institute, Section of Endocrine Surgery, Karolinska University Hospital, Stockholm, Sweden; 4 National Registry Centre, Skane University Hospital, Lund, Sweden; 5 Department of Internal Medicine, Helsingborg Hospital, Helsingborg, Sweden; 6 Section of Endocrine and Sarcoma Surgery, Department of Surgery, Skane University Hospital, Lund, Sweden; 7 Swedish Renal Registry, Ryhov Hospital, Jönköping, Sweden; 8 Scandinavian Quality Register for Thyroid Parathyroid and Adrenal Surgery, Helsingborg, Sweden

**Keywords:** End stage renal disease, Dialysis, Renal transplantation, Secondary hyperparathyroidism, Parathyroidectomy

## Abstract

**Background:**

Many patients on renal replacement therapy (RRT) require parathyroidectomy (PTX). Trends and current rates of PTX on a national level are not known. Furthermore, it is not completely clear which factors influence rates of PTX. Thus, our aim was to investigate the incidence, regional distribution and factors associated with PTX as well as possible temporal changes, in the Swedish RRT population.

**Methods:**

From the Swedish Renal Registry we extracted data on 20 015 patients on RRT between 1991 and 2009. In these, 679 incident PTX (3.4%) were identified by linkage with the National Inpatient Registry, and the Scandinavian Quality Registry for Thyroid Parathyroid and Adrenal Surgery. Poisson models were used to estimate rates per calendar year, adjusted for risk factors such as gender, age, time with renal transplant, and underlying cause of renal disease.

**Results:**

The PTX rate was 8.8/1 000 person-years. There was a significant increase 2001–2004 after which the rate fell, as compared with year 2000. Female gender, non-diabetic cause of renal disease and age between 40–55 were all associated with an increased frequency of PTX.

**Conclusion:**

The rise in PTX rates after year 2000 might reflect increasing awareness of the potential benefits of PTX. The introduction of calcimimetics and paricalcitol might explain the decreased rate after 2005.

## Background

Secondary hyperparathyroidism (sHPT) is a common complication of chronic kidney disease. Parathyroidectomy (PTX) is necessary in a substantial number of patients [1-4].

Previous studies reported wide variations in incidence of PTX over time [1-3,5] Younger age, female gender, absence of diabetes, longer time on dialysis [1,2,4,5] and lack of transplant [4] were all associated with an increased risk of PTX. However, as far as we know, no study has as yet investigated risk factors and rates of PTX in a country’s entire population of patients on renal replacement therapy (RRT), comprising peritoneal, hemodialysis and transplant patients, after the introduction of current medical therapy for sHPT. Our aim was to investigate the incidence, regional distribution and factors associated with PTX as well as possible temporal changes, in the Swedish dialysis and transplant population.

## Methods

This study included data from three prospective, population based, national registries. The Swedish Renal Registry, SRR [6], provided the patient cohort, whereas PTXs were identified in the Swedish National Inpatient Registry (SNIR), run by the Swedish National Board of Health and Welfare [7] and the Scandinavian Quality Register for Thyroid Parathyroid and Adrenal Surgery (SQRTPA), [8].

### Ethical approval

The study was approved by the Regional Ethics Committee of Lund, DNR 2010/483.

### Patient cohort

All patients included in the SRR between 1^st^Jan 1991 and 31^st^dec 2009 (n = 20,056) were eligible for the present study. The SRR is a national web-based quality registry for patients on maintenance RRT [9].

Out of 20,056 eligible patients, we excluded 13 patients due to errors in reporting. Thus, the patient cohort consisted of 20,043 patients.

The pharmaceutical company Amgen (Solna, Sweden) provided aggregate data on the sales of cinacalcet (Mimpara®) per calendar year in Sweden.

### Parathyroidectomy data

PTX was identified through linkage between the SRR cohort and SNIR, and between the SRR and the (SQRTPA), by using the national personal identification number.

SNIR has been validated, with overall good to excellent results regarding accuracy of coding [10].

A total of 954 PTX were identified by the following in-hospital codes within the admission data: extirpation of parathyroid gland, BBA30 *or* 0851, subtotal parathyroidectomy, BBA40 *or* 0852, total parathyroidectomy, *BBA50 or 0853*, implantation or transplantation of parathyroid gland, BBA70 *or* 0870, and any other operation on parathyroid gland*,* BBA99 *or* 0898.

SQRTPA was launched in 2004 and the coverage 2010 was 90%. In general, less than five percent of patients were not registered or incorrectly registered. In this registry, 128 PTX were identified. Of these, 126 were also found among the hospital admission data. A total of 956 PTX were identified. We defined an incident PTX as the first PTX occurring after registration in the SRR. Out of the 956 PTX, 229 were performed before registration in the SRR. Of the remaining 727 PTX, 41 were in patients with two operations, and 7 in patients with three operations, during the study period. Thus we found a total of 679 incident PTX.

### Determination of time-at-risk and follow-up

Start date was defined as date of registration in the SRR. Patients were censored at death, at PTX, when lost to follow-up, or at end of study. To be able to correct for the impact of renal transplantation on the risk for PTX, we used time spent with functioning transplant prior to censoring as a time-dependent covariate. The cumulative sum of time spent with a functioning transplant in all transplant episodes was calculated. The term zero time with a functioning graft was used for patients who never received a transplant or in whom the transplant failed to function at start. Time with transplant was divided into four categories: 0, 0–1 year, 1–3 years and >3 years. Due to censoring at time of entry a further 28 patients were excluded from statistical analysis.

### Statistical analysis

Means and standard deviations were used for continuous variables, and column percent for categorical variables. Time at risk in person-years was calculated for different categories of age, geographical region of treatment, cause of renal disease, and time with functioning graft, stratified by gender. The crude PTX rate was defined as the total number of PTX, divided by the total number of person- years. This was presented in various categories and stratified by gender. Poisson regression was used to estimate both unadjusted and adjusted incidence rate ratios. The model was adjusted for calendar year, gender, age, geographical region of treatment, cause of renal disease, and time with functioning graft. The PTX rate was divided into calendar years, with year 2000 as the reference year to evaluate the change in PTX incidence over time. The Poisson model goodness of fit was evaluated by comparison with negative binomial models. Unadjusted, aggregate sales of cinacalcet were graphically compared to unadjusted PTX rates for the years 2005–2009.

## Results

Patient characteristics at baseline are shown in Table 1. A total of 679 PTX were identified in 20,015 patients (3.4%). The mean follow-up (SD) time for the whole cohort was 3.9 (4.1) years. The overall unadjusted PTX rate in 77,624 person-years of follow-up was 8.8 incident PTX per 1000 person-years at risk (95% CI: 8.1-9.4), Table 2.

**Table 1:** Baseline characteristics in the Swedish dialysis- and transplant population

**Characteristics**	**Cohort**			**Parathyroidectomy**		
	**All**	**Male**	**Female**	**All**	**Male**	**Female**
	***n =*** **20,043**	***n =*** **12,903**	***n =*** **7140**	***n =*** **679**	***n =*** **333**	***n =*** **346**
		**64.4%**	**35.4%**		**49.0%**	**51.0%**
Age at baseline, years	62.8 (16.5)	63.1 (16.4)	62.2 (17.0)	50.3 (14.7)	50.2 (14.5)	50.4 (14.9)
Age at parathyroidectomy, years	-	-	-	54.3 (14.0)	54.3 (13.9)	54.3 (14.2)
Region						
North	*10.8*	*10.8*	*10.7*	*5.7*	*5.4*	*6.1*
Stockholm	*17.8*	*17.1*	*18.9*	*17.1*	*15.3*	*18.8*
South-east	*11.8*	*12.0*	*11.5*	*11.2*	*10.5*	*11.9*
South	*18.3*	*19.0*	*16.9*	*21.4*	*22.8*	*20.0*
Uppsala/Örebro	*23.4*	*23.2*	*23.7*	*21.7*	*24.0*	*19.4*
West	*17.6*	*17.5*	*17.8*	*22.5*	*21.3*	*23.7*
Abroad	*0.4*	*0.4*	*0.5*	*0.4*	*0.6*	*0.3*
Cause of renal disease						
Diabetes	*24.2*	*23.9*	*24.8*	*14.9*	*14.1*	*15.6*
Nephrosclerosis	*18.2*	*20.6*	*13.9*	*10.0*	*9.6*	*10.4*
Glomerulonephritis	*15.9*	*17.7*	*12.6*	*30.2*	*37.2*	*23.4*
Adult polycystic kidney disease	*7.8*	*6.5*	*10.2*	*14.7*	*12.3*	*17.1*
Pyelonephritis	*5.0*	*4.5*	*6.1*	*9.6*	*6.9*	*12.1*
Other	*28.8*	*26.8*	*32.4*	*20.6*	*19.8*	*21.4*

Values expressed as mean (SD); *column percent in italics.*

**Table 2:** Numbers and rates of parathyroidectomy, per 1000 person-years, in the Swedish dialysis- and transplant population

	**All**			**Male**			**Female**		
	***n***	**Person-years**	**Rate (95% CI)**	***n***	**Person-years**	**Rate (95% CI)**	***n***	**Person-years**	**Rate (95% CI)**
All	679	77,624	8.8 (8.1-9.4)	333	50,294	6.6 (5.9-7.4)	346	27,330	12.7 (11.4-14.0)
Age (years)									
0-40	89	22,076	4.0 (3.3-5.0)	43	14,535	2.6 (2.1-3.9)	46	7540	6.1 (4.6-8.14)
40-55	260	25,621	10.1 (9.0-11.5)	132	16,679	7.9 (6.7-9.4)	128	8943	14.3 (12.0-17.0)
55-70	211	17,906	11.8 (10.3-13.5)	99	11,599	8.5 (7.0-10.4)	112	6308	17.8 (14.8-21.4)
> 70	119	12,021	9.9 (8.3-11.9)	59	7481	7.9 (6.1-10.2)	60	4540	13.2 (10.3-17.0)
Region									
North	39	8115	4.8 (3.5-6.6)	18	5176	3.5 (2.2-5.2)	21	2940	7.1 (4.7-11.0)
Stockholm	116	14,337	8.0 (6.7-9.7)	51	8905	5.7 (4.3-7.5)	65	5432	12.0 (9.4-15.3)
South-east	76	8808	8.6 (6.9-10.8)	35	5792	6.0 (4.3-8.3)	41	3016	13.6 (10.0-18.5)
South	145	14,736	9.8 (8.4-11.6)	76	9994	7.6 (6.0-9.5)	69	4743	14.6 (11.5-18.4)
Uppsala/Örebro	147	17,267	8.5 (7.2-10.0)	80	11,211	7.1 (5.7-8.9)	67	6056	11.0 (8.7-14.0)
West	153	13,613	11.2 (9.6-13.2)	71	8706	8.1 (6.5-10.3)	82	4907	16.7 (13.5-20.8)
Abroad	3	747	4.0 (1.3-12.5)	2	510	3.9 (1.0-15.7)	1	237	4.2 (0.6-30.0)
Cause of renal disease									
Diabetes	101	15,984	6.3 (5.2-7.7)	47	10,385	4.5 (3.4-6.0)	54	5599	9.6 (7.4-12.6)
Nephrosclerosis	68	10,008	6.8 (5.4-8.6)	32	7262	4.4 (3.1-6.2)	36	2746	13.1 (9.5-18.2)
Glomerulonephritis	205	18,219	11.3 (9.8-12.9)	124	13,202	9.4 (7.9-11.2)	81	5018	16.1 (13.0-20.1)
Adult polycystic kidney disease	100	9481	10.5 (8.7-12.8)	41	5156	8.0 (5.9-10.8)	59	4325	13.6 (10.6-17.6)
Pyelonephritis	65	4905	13.3 (10.4-16.9)	23	2784	8.3 (5.5-12.4)	42	2121	19.8 (14.6-26.8)
Other	140	19,018	7.4 (6.2-8.7)	66	11,496	5.7 (4.5-7.3)	74	7522	9.8 (7.8-12.4)
Years with functioning transplant									
0	417	43,334	9.6 (8.7-10.6)	200	27,973	7.1 (6.2-8.2)	217	15,361	14.1 (12.4-16.1)
0 > and < 1	77	3056	25.2 (20.2-31.5)	39	1964	19.9 (14.5-27.2)	38	1091	34.8 (25.3-47.8)
≥ 1 and < 3	97	8427	11.5 (9.4-14.0)	47	5503	8.5 (6.4-11.4)	50	2924	17.1 (13.0-22.6)
≥ 3	88	22,808	3.9 (3.1-4.8)	47	14,854	3.2 (2.4-4.2)	41	7953	5.2 (3.8-7.0)

After adjustment, female gender, age group 40–55 years as compared with other age groups, and non-diabetic cause of end-stage renal disease were all associated with an increased risk for PTX, Table 3. Cumulative time with functioning renal transplant (more than one but less than three years, and more than three years as compared with zero time with functioning graft) was inversely associated with risk of PTX in the adjusted analysis, Table 3.

**Table 3:** Risk factors for parathyroidectomy, as incidence rate ratios (IRR), in the Swedish dialysis- and transplant population

**Factor**	**IRR (95% CI)**	
	**Unadjusted**	**Adjusted**
Gender		
Female	1.00	1.00
Male	0.53 (0.45-0.61)	0.53 (0.45-0.61)
Age (years)		
0-40	0.90 (0.71-1.12)	0.87 (0.69-1.09)
40-55	1.00	1.00
55-70	0.80 (0.67-0.96)	0.78 (0.65-0.94)
> 70	0.27 (0.21-0.34)	0.25 (0.19-0.32)
Region		
Stockholm	1.00	1.00
North	0.57 (0.40-0.83)	0.66 (0.46-0.94)
South-east	1.04 (0.78-1.39)	1.13 (0.84-1.51)
South	1.23 (0.97-1.58)	1.38 (1.08-1.77)
Uppsala/Örebro	1.00 (0.78-1.27)	1.19 (0.93-1.52)
West	1.37 (1.07-1.74)	1.49 (1.17-1.89)
Abroad	0.78 (0.25-2.47)	0.46 (0.15-1.44)
Cause of renal disease		
Diabetes	1.00	1.00
Nephrosclerosis	1.02 (0.75-1.39)	1.56 (1.14-2.13)
Glomerulonephritis	2.22 (1.75-2.80)	2.30 (1.80-2.94)
Adult polycystic kidney disease	2.17 (1.65-2.87)	1.88 (1.42-2.50)
Pyelonephritis	2.60 (1.90-3.56)	2.54 (1.85-3.48)
Other	1.22 (0.98-1.60)	1.30 (1.00-1.68)
Years with functioning transplant		
0	1.00	1.00
0 > and < 1	1.32 (1.03-1.68)	1.59 (1.23-2.04)
≥ 1 and < 3	3.22 (2.56-4.06)	0.71 (0.56-0.89)
≥ 3	1.00 (0.79-1.27)	0.24 (0.19-0.30)

There were few events and person-years in the first two years the SNR was operating, 1991 and 1992, hence incidence rate ratios for those years had wide confidence intervals. In the adjusted model there were no statistically significant differences in the PTX rate from 1993 to 2000 using year 2000 as a reference year, Table 4. The PTX rate was significantly higher in the years 2001 through 2004. In the subsequent years following year 2005, there was a decrease in the PTX rate, although this change did not reach statistical significance as compared with the reference year 2000, Figure 1, Table 4. Aggregate data on the sales of cinacalcet seemed to match the decreasing rate of PTX, Figure 2.

**Table 4:** Rates and incidence rate ratios (IRR) of parathyroidectomy per calendar year in the Swedish dialysis and transplant population

**Calendar year**	**Rate/1000 person-years**	**Unadjusted IRR**	Adjusted* IRR
	**(95% CI)**	**(95% CI)**	**(95% CI)**
1991	5.2 (1.3-20.8)	0.18 (0.04-0.74)	0.45 (0.11-1.87)
1992	0.9 (0.1-6.6)	0.06 (0.00-0.47)	0.08 (0.01-0.60)
1993	10.2 (6.3-16.4)	0.82 (0.46-1.46)	0.96 (0.54-1.72)
1994	11.9 (8.1-17.4)	1.13 (0.68-1.88)	1.18 (0.71-1.96)
1995	8.8 (5.9-13.3)	0.90 (0.54-1.54)	0.94 (0.56-1.60)
1996	12.1 (8.7-16.7)	1.30 (0.81-2.06)	1.33 (0.83-2.11)
1997	9.0 (6.3-12.9)	1.00 (0.61-1.62)	1.04 (0.64-1.69)
1998	7.0 (4.7-10.2)	0.82 (0.50-1.37)	0.83 (0.50-1.38)
1999	7.1 (4.1-10.3)	0.89 (0.54-1.45)	0.87 (0.53-1.43)
2000	8.0 (5.8-11.2)	1.00	1.00
2001	13.5 (10.6-17.3)	1.78 (1.18-2.7)	1.75 (1.16-2.64)
2002	13.2 (10.4-16.9)	1.75(1.16-2.64)	1.77 (1.17-2.67)
2003	12.4 (9.7-15.9)	1.69 (1.11-2.56)	1.70 (1.12-2.56)
2004	12.6 (9.9-16.0)	1.79 (1.19-2.70)	1.76 (1.17-2.65)
2005	10.3 (8.0-13.3)	1.45 (0.95-2.20)	1.47 (0.97-2.24)
2006	4.4 (3.0-6.4)	0.64 (0.39-1.07)	0.64 (0.39-1.07)
2007	5.4 (3.9-7.6)	0.81 (0.51-1.31)	0.82 (0.51-1.31)
2008	5.8 (4.2-8.0)	0.88 (0.56-1.40)	0.91 (0.57-1.44)
2009	5.7 (4.1-7.8)	0.88 (0.56-1.40)	0.90 (0.57-1.42)

*Adjusted for gender, age at baseline (in categories), cause of renal disease, region, years with functioning renal transplant (in categories).

**Figure 1:**
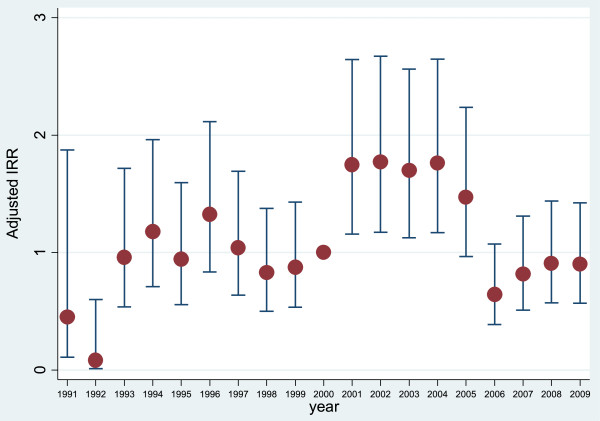
PTX incidence rate ratio, adjusted for gender, age (in categories), region, cause of renal disease and time with functioning renal transplant (in categories).

**Figure 2:**
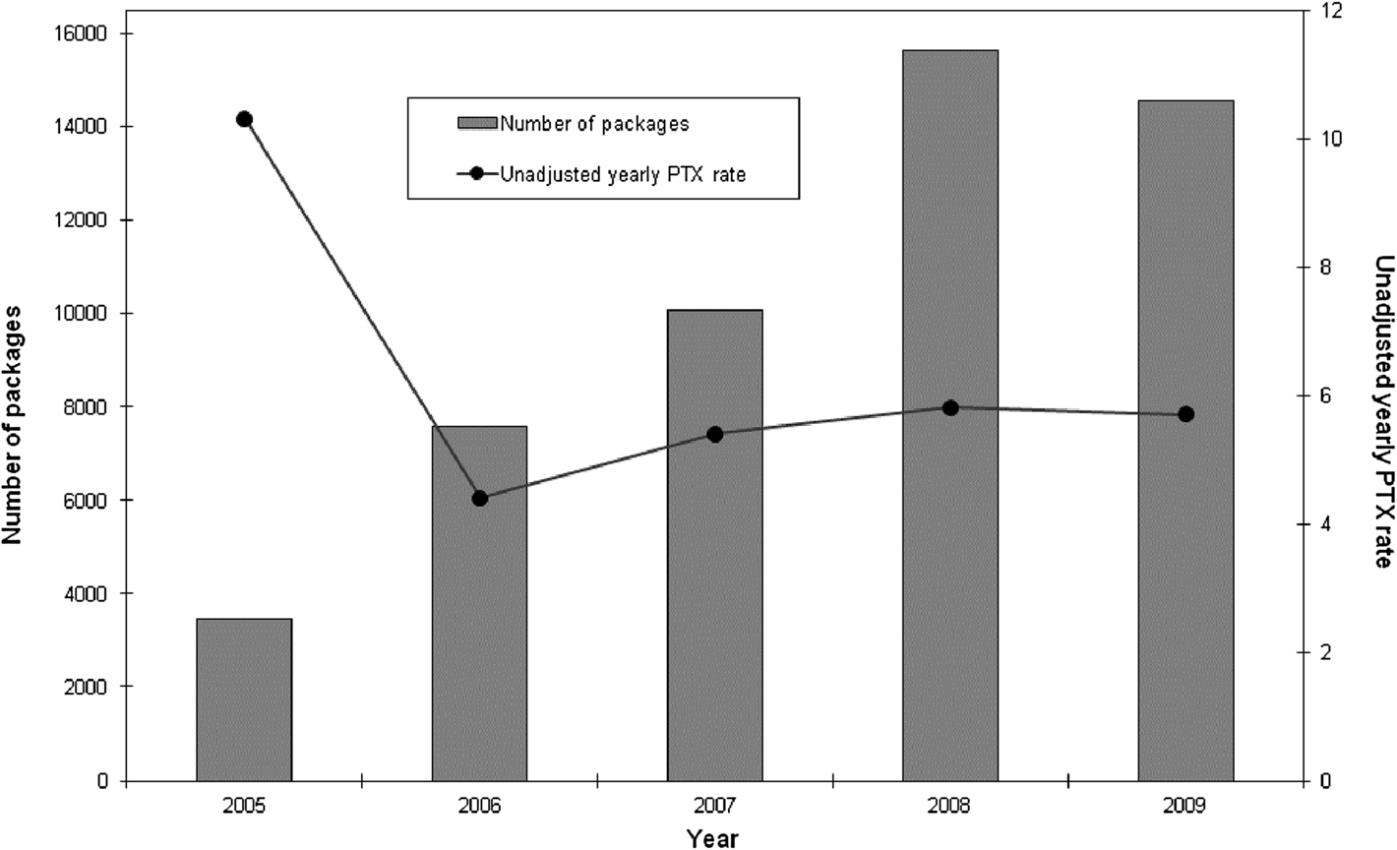
Aggregated sales of cinacalcet and yearly rates of PTX/1000 person-years at risk between 2005 and 2009.

## Discussion

In the present study, risk factors for PTX were female gender, age 40–55 years, and non-diabetic cause of renal disease. In the adjusted model, having had a functioning renal graft for less than one year was also associated with increased risk of PTX, whereas having had a functioning renal graft for more than one year was associated with decreased risk, compared with zero time with functioning graft.

The increased risk of PTX in women, patients in younger middle age and non-diabetics is congruent with results from previous studies [1-4]. The regional distribution of PTX was heterogeneous with the lowest rate in the Northern region. We believe, as was also suggested by previous authors, that lower access to specialist nephrology might account for these regional differences [2]. Having had a functioning renal transplant for more than a year was associated with a decreased risk of PTX in the adjusted analysis. This is to be expected, given that renal transplantation usually ameliorates sHPT [11].

In the present study the overall unadjusted PTX rate was 8.8 (95% CI, 8.1-9.4) per 1000 person-years, which is in line with previous investigations, reporting an incidence between 4.3 and 15.2/1000 person-years [1-5,12].

We found no significant differences in the PTX incidence during the period 1993 to 2000 in comparison with the reference year 2000. However, from 2001 to 2004 the rate increased significantly, even after adjusting for patient characteristics, and showed a tendency to decrease from 2005 onwards. Our results are similar to the most recent study reporting change over time of PTX in sHPT by Li *et. al*. They found that the adjusted PTX rate increased in 1998, peaked in 2002 and decreased through 2005 with a new increase in 2006 and 2007 [3]. Malberti *et. al* showed a relatively constant PTX rate throughout the study period of 1983 and 1996 [5]. Kestenbaum *et. al* reported a slight rise in adjusted PTX rates between 1990 and 1995 with a subsequent decline of 30% in 1999 [1]. Foley *et. al* reported a progressive decline from 1992 to 1998 with an increase in 2002 [2].

There are several possible explanations for this reported diversity in PTX rates. Firstly, the studies cover a large span of time from 1971 to 2007 with different observational and comparison periods. Secondly, the patient cohorts studied are heterogenous, often comprising subgroups of RRT patients [1-4].

Our study comprises all RRT patients in Sweden and covers the period before and after key publications on the consequences of sHPT on mortality and cardiovascular risk [13-16] and after the publication of the KDOQI guidelines for PTX [17]. The increase in PTX rates after 2000 might be a consequence of these reports. After 2005 the PTX rate fell and remained lower throughout the study period. This decrease was associated in time with the introduction of paracalcitol in December 2004 and calcimimetics in 2005 in Sweden. Changes in PTX rates coincided with changes in the aggregate sale of cinacalcet during this period (Figure 2).

A limitation of the present study is the lack of biochemical data, such as levels of parathyroid hormone, and of information on medical treatment. Strengths include the high quality of the Swedish Renal Registry, with an almost 100% nation wide coverage and a data reporting incidence of 95% [6]. In addition, our follow-up period comprises different eras of treatment availability and modality. The fact that this study is based on an entire population with a wide geographical distribution within the country is to our knowledge a unique situation. We also believe that external validity was increased by including all patients on RRT, irrespective of treatment modality. The linkage of three nation-wide registries with broad and accurate coverage further adds to the validity of the present results.

## Conclusion

PTX rates differed over time in Swedish patients on RRT. This might reflect increasing awareness of the potential benefits of treating sHPT, and it is possible that the fall in rates from 2005 was caused by the introduction of calcimimetics. Female gender, non-diabetic cause of renal disease and age group 40–45 compared with younger and older patients, and cumulative time with functioning graft less than one year were all associated with higher risk for PTX.

## Abbreviations

RRT: Renal replacement therapy; PTX: Parathyroidectomy; sHPT: Seconcary hyperparathyroidism; SRR: Swedish renal registry; SNIR: Swedish national inpatient registry; SQRTPA: Scandinavian quality register for thyroid parathyroid and adrenal surgery; KDOQI: Kidney disease outcomes quality initiative.

## Competing interests

The authors declare that they have no competing interests.

## Authors’ contributions

SA: Conception and design, analysis and interpretation of the data, drafting and revising the article, interpretation of the results, providing intellectual content of critical importance. Final approval. NC: Interpretation of the results, revising the article, providing intellectual content of critical importance. Final approval. GS: Revising the article, providing intellectual content of critical importance. Final approval. BR: Chairperson of the Swedish Renal Registry and responsible for the Registry’s validity, providing intellectual content of critical importance. Final approval. ER: Chairperson of the Scandinavian Quality Registry for Thyroid and Parathyroid Surgery and responsible for this Registry’s validity, interpretation of the results, revising the article, providing intellectual content of critical importance. Final approval. PW: Responsible for the design of the statistical analyses. Final approval. RR: Responsible for the design and execution of the statistical analyses. Final approval. KGP: General secreterary of the Swedish Renal Registry with operative responsibility for the validity of the Registry’s data, providing intellectual content of critical importance. Final approval. MA: Conception and design, responsible for the epidemiological design, analysis and interpretation of the data, drafting and revising, providing intellectual content of critical importance. Final approval.

## Pre-publication history

The pre-publication history for this paper can be accessed here:

http://www.biomedcentral.com/1471-2369/15/75/prepub
